# Is local review of positron emission tomography scans sufficient in diffuse large B‐cell lymphoma clinical trials? A CALGB 50303 analysis

**DOI:** 10.1002/cam4.5628

**Published:** 2023-02-17

**Authors:** Pallawi Torka, Levi D. Pederson, Michael V. Knopp, David Poon, Jun Zhang, Brad S. Kahl, Howard R. Higley, Gary Kelloff, Jonathan W. Friedberg, Lawrence H. Schwartz, Wyndham H. Wilson, John P. Leonard, Nancy L. Bartlett, Heiko Schöder, Amy S. Ruppert

**Affiliations:** ^1^ Department of Medicine Roswell Park Cancer Comprehensive Cancer Center Buffalo New York USA; ^2^ Alliance Statistics and Data Management Center Mayo Clinic Rochester Minnesota USA; ^3^ Department of Radiology The Ohio State University Columbus Ohio USA; ^4^ Department of Medicine Washington University School of Medicine St. Louis Missouri USA; ^5^ CCS Associates, Inc. San Jose California USA; ^6^ Division of Cancer Treatment and Diagnosis National Cancer Institute, National Institutes of Health Rockville Maryland USA; ^7^ Department of Medicine University of Rochester Medical Center Rochester New York USA; ^8^ Department of Radiology Columbia University Medical Center New York New York USA; ^9^ Lymphoid Malignancies Branch, National Cancer Institute, National Institutes of Health Rockville Maryland USA; ^10^ Department of Medicine Weill Cornell Medical College New York New York USA; ^11^ Department of Radiology Memorial Sloan Kettering Cancer Center New York New York USA; ^12^ Department of Internal Medicine The Ohio State University Columbus Ohio USA

**Keywords:** Deauville 5‐PS, interim PET, International Harmonization Project criteria, visual scoring system, ΔSUV

## Abstract

**Background:**

Quantitative methods of Fluorodeoxyglucose Positron Emission Tomography (FDG‐PET) interpretation, including the percent change in FDG uptake from baseline (ΔSUV), are under investigation in lymphoma to overcome challenges associated with visual scoring systems (VSS) such as the Deauville 5‐point scale (5‐PS).

**Methods:**

In CALGB 50303, patients with DLBCL received frontline R‐CHOP or DA‐EPOCH‐R, and although there were no significant associations between interim PET responses assessed centrally after cycle 2 (iPET) using 5‐PS with progression‐free survival (PFS) or overall survival (OS), there were significant associations between central determinations of iPET ∆SUV with PFS/OS. In this patient cohort, we retrospectively compared local vs central iPET readings and evaluated associations between local imaging data and survival outcomes.

**Results:**

Agreement between local and central review was moderate (kappa = 0.53) for VSS and high (kappa = 0.81) for ∆SUV categories (<66% vs. ≥66%). ∆SUV ≥66% at iPET was significantly associated with PFS (*p* = 0.03) and OS (*p* = 0.002), but VSS was not. Associations with PFS/OS when applying local review vs central review were comparable.

**Conclusions:**

These data suggest that local PET interpretation for response determination may be acceptable in clinical trials. Our findings also highlight limitations of VSS and call for incorporation of more objective measures of response assessment in clinical trials.

## Graphical abstract

In this retrospective analysis of CALGB 50303 study, Torka et al. found that associations with PFS and OS when applying local review versus central review of interim PET (iPET) were comparable? SUV = 66% at iPET was associated with PFS and OS, but visual scoring systems (VSS) were not, highlighting the limitations of VSS.

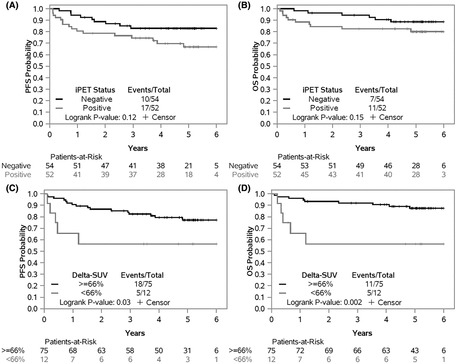

## 1 INTRODUCTION

In patients with diffuse large B‐cell lymphoma (DLBCL), interim fluorodeoxyglucose (FDG) positron emission tomography (PET)–computed tomography (CT) imaging (iPET) is used to assess early treatment response, and PET‐CT at the end of treatment (EOT PET) is used to establish remission status. Visual scoring systems (VSS) including the International Harmonization Project criteria (IHPC)^1^ and more recently, the Deauville 5‐point scale (5‐PS),^2^ have been widely used in clinical trials for standardized interpretation of scans in patients with FDG‐avid histologies.^3^ While the negative predictive value of FDG‐PET at iPET and EOT is high in most studies, the positive predictive value is low due to false positives caused by inflammation and tumor necrosis.^4^, ^5^ This limitation, along with poor interobserver reproducibility, has hampered our ability to successfully utilize iPET‐adapted therapy in advanced stage DLBCL to date.^4^, ^6^, ^7^ To overcome challenges of VSS, quantitative methods, such as percent change in FDG uptake from baseline (ΔSUV), have been evaluated.^8^, ^9^, ^10^ The phase three Cancer and Leukemia Group B (CALGB) 50,303 study compared DA‐EPOCH‐R with standard R‐CHOP as frontline therapy for DLBCL and demonstrated lack of improved outcomes with the more toxic DA‐EPOCH‐R regimen.^11^ A sub‐analysis of 158 patients with central imaging data failed to show an association between iPET (after cycle 2) response using 5‐PS and progression‐free survival (PFS) or overall survival (OS) but did show a significant association between iPET ∆SUV and PFS/OS.^10^ CALGB is now part of the Alliance for Clinical Trials in Oncology.

As central review of FDG‐PET is not applicable in routine practice, it is important to assess the prognostic impact of VSS and ΔSUV based on local PET interpretation. If local and central determinations were comparable, local assessments could obviate the additional time, effort, and expense of central assessments in clinical trials. With little data comparing local and central determinations in DLBCL,^12^, ^13^, ^14^, ^15^ we retrospectively compared local versus central iPET readings in patients on CALGB 50303.

## 2 METHODS

In CALGB 50303 trial, patients could consent to an optional imaging substudy (CALGB 580603), which included FDG‐PET at baseline (≤30 days of therapy), 17–21 days post cycle 2 (0–4 days before cycle 3), and 4–8 weeks after completion of cycle 6. Interim scans were collected for investigational purposes and treating physicians were blinded to results unless local nuclear medicine physicians noted an urgent finding. Scans were not used to alter therapy. This study was approved by the Institutional Review Boards of all participating institutions (master protocol number CALGB 50303/CTSU 50303/NCT00118209) with written informed consent obtained from each participant and/or their legal representative, as appropriate.

Technical details and quality measures for PET imaging were described previously.^10^ Reviewer training and data transfer details are in Appendix S1. For interim and EOT scans, IHPC was the standard of care VSS at the time of study conduct and used for local response interpretations, whereas 5‐PS was used for central response interpretations performed after study completion (Table 1A). IHPC/5‐PS scores 0–2/1–3 were defined prospectively as negative and scores 3–4/4–5 as positive.^1^, ^3^ To address differences in VSS, data were also analyzed by retrospectively regrouping 5‐PS scores of 3–5 as positive. The percent change in FDG uptake was defined as the difference between the highest SUV in any disease site from baseline to follow‐up, as a fraction of the former: ∆SUV = 100% × (baseline maxSUV − follow‐up maxSUV)/baseline maxSUV. ∆SUV was analyzed using a predefined cut‐point of 66%, with ∆SUV ≥ 66% corresponding to a high reduction in FDG uptake.^8^, ^9^, ^10^ PFS and OS distributions were landmarked at iPET, estimated using the Kaplan–Meier method and compared between groups using two‐sided log‐rank tests. Cox proportional hazards models were used to correlate FDG PET measures with PFS and OS when adjusting for the International Prognostic Index (IPI) risk group. Statistical significance was declared with *p* < 0.05.

**TABLE 1:** Comparison of local and central iPET by VSS and % decrease in maxSUV (ΔSUV). (A) Comparison of International Harmonization Project criteria (IHPC) that were applied for local iPET response determination and Deauville 5‐point scale (5‐PS) that was applied for central iPET response determination. (B) Comparison of Local and Central iPET Status after Cycle 2 by VSS (*n* = 106). (C) Comparison of Local and Central % Decrease in maxSUV on iPET after Cycle 2 (*n* = 87). (D) SUV data for the 4 patients with discrepant ΔSUV adjudication between central and local reads (2 in each direction) at iPET after 2 cycles

(A) PET response	IHPC		5‐PS	
Negative	0	No abnormal activity (tumor cold compared with background)	1	No uptake above background
	1	Minimal activity (tumor less than background)	2	Slight uptake, but equal to or below blood pool (mediastinum)
	2	Equivocal (tumor=background)	3	Uptake above mediastinal, but below or equal to uptake in the liver
Positive	3	Moderately increased activity (tumor greater than background for lesions < 2 cm; tumor greater than mediastinal blood pool for lesions > 2 cm)	4	Uptake slightly to moderately higher than liver
	4	Markedly increased activity (tumor much greater than background) New foci of FDG activity judged to represent lymphoma	5	Markedly increased uptake or any new lesions

(B) Local determination	Central determination		Total
	Positive	Negative	
Positive	32	20	52
Negative	5	49	54
Total	37	69	106
*Agreement and performance measures using central reads as the reference*. Overall Agreement: (32 + 49)/106 = 76.4% Positive Predictive Value: 32 / 52 = 61.5% Negative Predictive Value: 49 / 54 = 90.7% Sensitivity: 32 / 37 = 86.5% Specificity: 49 / 69 = 71.0% Kappa Statistic: 0.53 (95% CI: 0.37–0.68)			

(C) Local determination	Central determination		Total
	ΔSUV > 66%	ΔSUV < 66%	
ΔSUV > 66%	73	2	75
ΔSUV < 66%	2	10	12
Total	75	12	87
*Agreement and performance measures using central reads as the reference*. Overall Agreement: (73 + 10)/87 = 95.4% Positive Predictive Value: 73 / 75 = 97.3% Negative Predictive Value: 10 / 12 = 83.3% Sensitivity: 73 / 75 = 97.3% Specificity: 10 / 12 = 83.3% Kappa Statistic: 0.81 (95% CI: 0.62–0.99)			

(D) Local reads				Central reads			
Baseline maxSUV	Cycle 2 maxSUV	Delta‐SUV (%)	Delta‐SUV Group (%)	Baseline maxSUV	Cycle 2 maxSUV	Delta‐SUV (%)	Delta‐SUV Group (%)
24.5	9.5	61.2	<66	24.5	7.3	70.2	>66
9.7	3.4	64.9	<66	9.7	1.5	84.5	>66
33.7	4.2	87.5	>66	33.7	12.4	63.2	<66
37	8.5	77.0	>66	36.9	12.9	65.0	<66

## 3 RESULTS

Of 524 patients enrolled on CALGB 50303, 169 consented to the FDG‐PET substudy. Of 158 patients included in central imaging analyses,^10^ 106 had local VSS results and 87 had local ∆SUV results at iPET. There were no significant differences between baseline characteristics of patients included in the substudy versus the parent trial.^7^

### 3.1 Comparison of local versus central reads

In 106 patients with VSS results, 52 (49.1%) were iPET^+^ by local review and 37 (34.9%) by central review (Table 1B). Agreement in local and central review was moderate (kappa = 0.53), occurring in 81 patients (76.4%; 32 iPET^+^ and 49 iPET^−^). Disagreement occurred in 25 patients, 20 with local iPET^+^ but central iPET^−^ disease and 5 with local iPET^−^ but central iPET^+^ disease. When 5‐PS score of three was considered positive in an effort to more closely match the IHPC and 5‐PS scales, the agreement between local and central review was fair (kappa = 0.36), occurring in 72 patients (67.9%; 40 iPET^+^ and 32 iPET^−^; Figure S1).

Median ∆SUV was 84.6% (range: −3.0% to 95.9%) by local review and 85.1% (range: −34.9% to 95.8%) by central review. ∆SUV was <66% in 12 (13.8%) patients by local review and by central review (Table 1C). Agreement of ∆SUV in local and central review was high (kappa = 0.81), occurring in 83 patients (95.4%; 10 with ∆SUV <66%, 73 with ∆SUV ≥66%). Disagreement occurred in four patients, two in each direction (Table 1D).

### 3.2 Association of PET responses with survival outcomes

**FIGURE 1:**
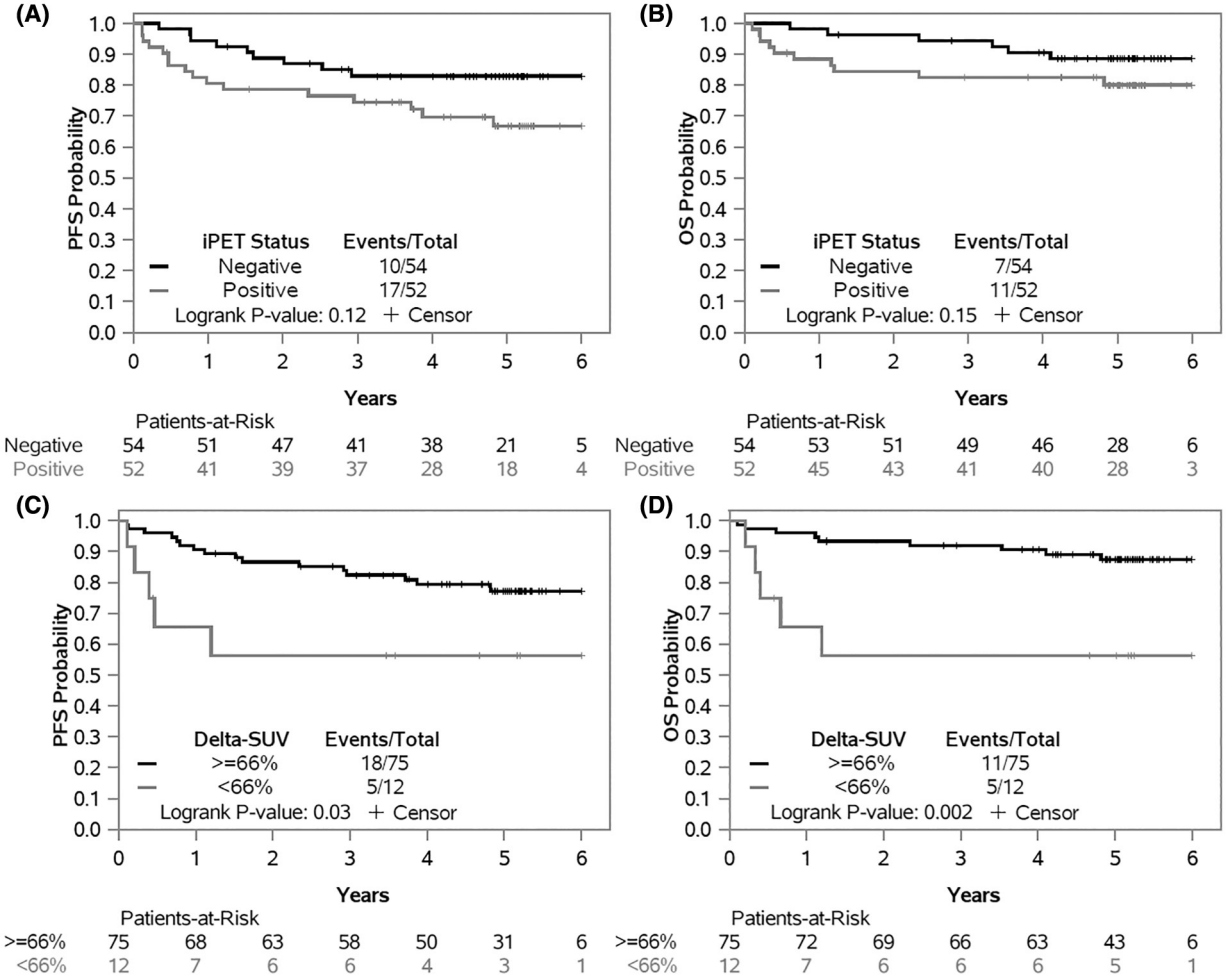
Progression free survival (PFS) and Overall survival (OS) landmarked at iPET, according to iPET status by IHPC (negative/positive) (A and B) and by prespecified ΔSUV groups (C and D) using local PET interpretation.

Using local data and the prospectively defined iPET^−^ (IHPC/5‐PS scores 0–2/1–3) and iPET^+^ (IHPC/5‐PS scores 3–4/4–5) categories, PFS and OS estimates were numerically lower in patients with iPET^+^ versus iPET^−^ disease but not statistically significant (*p* = 0.12 and *p* = 0.15; Figure 1A,B). Two‐year estimates for PFS were 79% (95% CI 68–91%) and 89% (95% CI 81–98%), and 2‐year estimates for OS were 84% (95% CI 75–95%) and 96% (95% CI 91–100%) for local iPET^+^ and iPET^−^, respectively. In contrast, ∆SUV groups by local review were significantly associated with PFS and OS (*p* = 0.03 and *p* = 0.002; Figure 1C,D). Two‐year PFS estimates were 56% (95% CI 34–94%) and 87% (95% CI 79–95%) and 2‐year OS estimates were 56% (95% CI 34–94%) and 93% (95% CI 88–99%) for ∆SUV <66% and ∆SUV ≥66%, respectively. When adjusting for IPI risk group, ∆SUV was moderately associated (*p* = 0.06) with PFS and remained a significant prognostic factor for OS (*p* = 0.005). As reference, PFS and OS curves using central data for this patient subset are provided in Figure S2.

## 4 DISCUSSION

In this retrospective study, our data showed moderate agreement between iPET local and central reads using VSS, with most discrepancies arising from a local iPET^+^ result and central iPET^−^ result. This is not surprising since the criteria for IHPC score three (positive) overlaps with 5‐PS score three (negative). The discrepancy between iPET interpretation remained when 5‐PS score three was considered positive and data were reanalyzed, suggesting that it was not merely a result of using different VSS. Regardless, it is important to note that neither VSS identified a subgroup of patients with very poor outcomes. It is significant to note that agreement in ∆SUV of <66% versus ≥66% was high between local and central review. Similar to previous analyses using central data, associations with PFS and OS using local data for ∆SUV were significant and stronger than VSS.^10^, ^16^

Our observation is consistent with previous studies that have shown that while iPET based response using VSS has utility in predicting outcomes in DLBCL, a more robust association is noted when ∆SUV is incorporated in the response adjudication.^8^, ^9^, ^16^, ^17^ In a systematic review of 19 studies comprising 2366 patients, the negative predictive value (NPV) of iPET by VSS for progression within 2 years was high (64%–95%), however the sensitivity (33%–87%), specificity (49%–94%), and positive predictive values (20%–74%) were variable.^18^ A recent meta‐analysis that reviewed 1692 iPET results from patients with DLBCL treated with R‐CHOP in the European PETRA database showed that iPET was predictive of 2‐year PFS in all International Prognostic Index (IPI) risk groups.^17^ This study however also raised questions around the optimal categorization of iPET positivity. Additionally, though optimal timing of iPET remains unclear, this study showed similar positive predictive value of iPET when conducted after cycles 2 or 4.

Another factor that hinders use of iPET in real‐time decision making in clinical trials is the need for central review for response adjudication which can lead to delays in subsequent treatment allotment. Blinded independent review committees in clinical trials aim to improve objectivity and reliability of data that might be otherwise subject to observer bias and variability^12^, ^13^, ^14^, ^19^, ^20^; however, even reviews by expert nuclear medicine physicians demonstrated only moderate consistency.^21^ Our data suggest that in the current era, local interpretation of FDG‐PET, in particular using objective parameters such as ∆SUV, is reliable for patients with DLBCL treated with standard chemoimmunotherapy, and its association with outcomes is comparable to central reads. Whether local review of FDG‐PET is comparable to central reads in patients receiving immunomodulating agents, immune checkpoint inhibitors or cell therapies needs to be studied. Occasionally these approaches cause “pseudoprogression,” likely related to recruitment of immune cells to the disease site, making accurate interpretation of scans challenging. Immune‐related response criteria were proposed in 2017 (RECIL 2017) to address such situations.^22^ Clinical trials using these agents would benefit from continued central review for response adjudication.

Our data has several limitations, including the use of IHPC versus 5‐PC for local and central VSS, respectively, that has been addressed above. Additionally, since this was a retrospective analysis, we could not thoroughly examine reasons for disagreements between local and central reads (e.g., inconsistency between target lesions). Lastly, small numbers of events limited our ability to conduct meaningful subgroup analyses.

In conclusion, our study highlights the limitations of using VSS and supports the use of more objective measures such as ΔSUV for response assessment. It also calls for the reassessment of the need for labor‐ and cost‐intensive central reviews as a routine in trials. Given 15%–20% of patients with DLBCL in a metabolic CR will relapse, newer imaging parameters such as ΔSUV or total tumor metabolic volume, or non‐imaging methods such as ctDNA, should be explored as biomarkers, especially when risk‐adapted therapeutic decisions are under investigation.

## AUTHOR CONTRIBUTIONS

**Pallawi Torka:** Formal analysis (equal); writing – original draft (equal); writing – review and editing (equal). **Levi D. Pederson:** Data curation (equal); formal analysis (equal); investigation (equal); writing – original draft (equal); writing – review and editing (equal). **Michael V. Knopp:** Data curation (equal); resources (equal); writing – review and editing (equal). **David Poon:** Data curation (equal); resources (equal); writing – review and editing (equal). **Jun Zhang:** Data curation (equal); resources (equal); writing – review and editing (equal). **Brad S. Kahl:** Data curation (equal); investigation (equal); resources (equal); writing – review and editing (equal). **Howard R. Higley:** Data curation (equal); project administration (equal); resources (equal); writing – review and editing (equal). **Gary Kelloff:** Data curation (equal); investigation (equal); resources (equal); writing – review and editing (equal). **Jonathan W. Friedberg:** Data curation (equal); investigation (equal); methodology (equal); resources (equal); writing – review and editing (equal). **Lawrence H. Schwartz:** Data curation (equal); investigation (equal); resources (equal); writing – review and editing (equal). **Wyndham H. Wilson:** Conceptualization (equal); data curation (equal); investigation (equal); methodology (equal); resources (equal); writing – review and editing (equal). **John P. Leonard:** Conceptualization (equal); data curation (equal); formal analysis (equal); funding acquisition (equal); investigation (equal); methodology (equal); project administration (equal); resources (equal); supervision (equal); writing – original draft (equal); writing – review and editing (equal). **Nancy L. Bartlett:** Conceptualization (equal); data curation (equal); formal analysis (equal); funding acquisition (equal); investigation (equal); methodology (equal); project administration (equal); resources (equal); supervision (equal); writing – original draft (equal); writing – review and editing (equal). **Heiko Schöder:** Conceptualization (equal); data curation (equal); formal analysis (equal); funding acquisition (equal); investigation (equal); methodology (equal); project administration (equal); resources (equal); supervision (equal); writing – original draft (equal); writing – review and editing (equal). **Amy S. Ruppert:** Data curation (equal); formal analysis (equal); investigation (equal); methodology (equal); project administration (equal); resources (equal); supervision (equal); writing – original draft (equal); writing – review and editing (equal).

## CONFLICT OF INTEREST

**Pallawi Torka**: Consulting advice: TG therapeutics, ADC therapeutics, Genentech, Kura Oncology. **Brad S. Kahl**: Consulting advice: Genentech, MTEM, MEI, Pharmacyclics, ADCT, Abbvie, Kite, BMS, Beigene, AstraZeneca, TG therapeutics, Epizyme, Takeda, Hutchmed, Genmab. Research Support: Genentech, AstraZeneca, Beigene. **Howard R Higley**: HRH was a former employee (now retired) of CCS Associates, a contract research organization supporting the NCI and FNIH, when the work described in this manuscript was performed. At that time, he had no further conflicts or disclosures to report. **Lawrence H. Schwartz**: DSMB/endpoint committee payable to institution: Merck, BMS, Regeneron. Research support: JNJ. Patents planned, issued or pending: Image segmentation licensed to Varian, payable to institution. **John P. Leonard**: Consulting advice: Abbvie, Astellas, AstraZeneca, Bayer, Beigene, BMS, Calithera, Constellation, Eisai, Lilly, Epizyme, Genmab, Grail, Incyte, Janssen, Karyopharm, Merck, Mustang Bio, Pfizer, Roche/Genentech, Second Genome, Sutro. Research support: Genentech, Janssen. **Amy S. Ruppert**: ASR is currently employed by Eli Lilly and Company, though contributions to the work described in this manuscript occurred during employment with The Ohio State University which ended as of January 2022; served on an independent DSMB for Telios Pharma. **Levi D. Pederson**, **Michael V. Knopp**, **David Poon**, **Jun Zhang**, **Gary Kelloff**, **Jonathan W. Friedberg**, **Wyndham H. Wilson**, **Nancy L. Bartlett** and **Heiko Schöder**: none.

## Supporting information


Appendix S1

## Data Availability

The data that support the findings of this study are available from Alliance for Clinical Trials in Oncology upon request.

## References

[1] Juweid ME , Stroobants S , Hoekstra OS , et al. Use of positron emission tomography for response assessment of lymphoma: consensus of the imaging Subcommittee of International Harmonization Project in lymphoma. J Clin Oncol. 2007;25(5):571‐578. 17242397 10.1200/JCO.2006.08.2305

[2] Meignan M , Gallamini A , Meignan M , Gallamini A , Haioun C . Report on the first international workshop on interim‐PET scan in lymphoma. Leuk Lymphoma. 2009;50(8):1257‐1260. 19544140 10.1080/10428190903040048

[3] Cheson BD , Fisher RI , Barrington SF , et al. Recommendations for initial evaluation, staging, and response assessment of Hodgkin and non‐Hodgkin lymphoma: the Lugano classification. J Clin Oncol. 2014;32(27):3059‐3068. 25113753 10.1200/JCO.2013.54.8800 PMC4979083

[4] Moskowitz CH , Schöder H , Teruya‐Feldstein J , et al. Risk‐adapted dose‐dense immunochemotherapy determined by interim FDG‐PET in advanced‐stage diffuse large B‐cell lymphoma. J Clin Oncol. 2010;28(11):1896‐1903. 20212248 10.1200/JCO.2009.26.5942 PMC3651601

[5] Han HS , Escalón MP , Hsiao B , Serafini A , Lossos IS . High incidence of false‐positive PET scans in patients with aggressive non‐Hodgkin's lymphoma treated with rituximab‐containing regimens. Ann Oncol. 2009;20(2):309‐318. 18842613 10.1093/annonc/mdn629 PMC2733066

[6] Swinnen LJ , Li H , Quon A , et al. Response‐adapted therapy for aggressive non‐Hodgkin's lymphomas based on early [18F] FDG‐PET scanning: ECOG‐ACRIN cancer research group study (E3404). Br J Haematol. 2015;170(1):56‐65. 25823885 10.1111/bjh.13389 PMC4696544

[7] Dührsen U , Müller S , Hertenstein B , et al. Positron emission tomography–guided therapy of aggressive non‐Hodgkin lymphomas (PETAL): a multicenter, randomized phase III trial. J Clin Oncol. 2018;36(20):2024‐2034. 29750632 10.1200/JCO.2017.76.8093

[8] Lin C , Itti E , Haioun C , et al. Early ^18^F‐FDG PET for prediction of prognosis in patients with diffuse large B‐cell lymphoma: SUV‐based assessment versus visual analysis. J Nucl Med. 2007;48(10):1626‐1632. 17873129 10.2967/jnumed.107.042093

[9] Casasnovas R‐O , Meignan M , Berriolo‐Riedinger A , et al. SUVmax reduction improves early prognosis value of interim positron emission tomography scans in diffuse large B‐cell lymphoma. Blood. 2011;118(1):37‐43. 21518924 10.1182/blood-2010-12-327767

[10] Schöder H , Polley MC , Knopp MV , et al. Prognostic value of interim FDG‐PET in diffuse large cell lymphoma: results from the CALGB 50303 clinical trial. Blood. 2020;135(25):2224‐2234. 32232481 10.1182/blood.2019003277 PMC7316220

[11] Bartlett NL , Wilson WH , Jung SH , et al. Dose‐adjusted EPOCH‐R compared with R‐CHOP as frontline therapy for diffuse large B‐cell lymphoma: clinical outcomes of the phase III intergroup trial Alliance/CALGB 50303. J Clin Oncol. 2019;37(21):1790‐1799. 30939090 10.1200/JCO.18.01994 PMC6774813

[12] Ansell SM , Armitage JO . Positron emission tomographic scans in lymphoma: convention and controversy. Mayo Clin Proc. 2012;87(6):571‐580. 22677077 10.1016/j.mayocp.2012.03.006 PMC3498383

[13] Zijlstra JM , Comans EF , van Lingen A , et al. FDG PET in lymphoma: the need for standardization of interpretation. An observer variation study. Nucl Med Commun. 2007;28(10):798‐803. 17728610 10.1097/MNM.0b013e3282eff2d5

[14] Meignan M , Itti E , Bardet S , et al. Development and application of a real‐time on‐line blinded independent central review of interim PET scans to determine treatment allocation in lymphoma trials. J Clin Oncol. 2009;27(16):2739‐2741. 10.1200/JCO.2009.22.4089 19398565

[15] Barrington SF , Qian W , Somer EJ , et al. Concordance between four European centres of PET reporting criteria designed for use in multicentre trials in Hodgkin lymphoma. Eur J Nucl Med Mol Imaging. 2010;37(10):1824‐1833. 20505930 10.1007/s00259-010-1490-5

[16] Itti E , Meignan M , Berriolo‐Riedinger A , et al. An international confirmatory study of the prognostic value of early PET/CT in diffuse large B‐cell lymphoma: comparison between Deauville criteria and ΔSUVmax. Eur J Nucl Med Mol Imaging. 2013;40(9):1312‐1320. 23649463 10.1007/s00259-013-2435-6

[17] Eertink JJ , Burggraaff CN , Heymans MW , et al. Optimal timing and criteria of interim PET in DLBCL: a comparative study of 1692 patients. Blood Adv. 2021;5(9):2375‐2384. 33944897 10.1182/bloodadvances.2021004467 PMC8114547

[18] Burggraaff CN , de Jong A , Hoekstra OS , et al. Predictive value of interim positron emission tomography in diffuse large B‐cell lymphoma: a systematic review and meta‐analysis. Eur J Nucl Med Mol Imaging. 2019;46(1):65‐79. 30141066 10.1007/s00259-018-4103-3 PMC6267696

[19] Amit O , Mannino F , Stone AM , et al. Blinded independent central review of progression in cancer clinical trials: results from a meta‐analysis. European Journal of Cancer. 2011;47(12):1772‐1778. 21429737 10.1016/j.ejca.2011.02.013

[20] Dancey JE , Dodd LE , Ford R , et al. Recommendations for the assessment of progression in randomised cancer treatment trials. European Journal of Cancer. 2009;45(2):281‐289. 19097775 10.1016/j.ejca.2008.10.042

[21] Horning SJ , Juweid ME , Schöder H , et al. Interim positron emission tomography scans in diffuse large B‐cell lymphoma: an independent expert nuclear medicine evaluation of the eastern cooperative oncology group E3404 study. Blood. 2010;115(4):775‐777. quiz 918. 19767508 10.1182/blood-2009-08-234351 PMC2815514

[22] Younes A , Hilden P , Coiffier B , et al. International working group consensus response evaluation criteria in lymphoma (RECIL 2017). Ann Oncol. 2017;28(7):1436‐1447. 28379322 10.1093/annonc/mdx097 PMC5834038

