# Supplementary material for: Is local review of positron emission tomography scans sufficient in diffuse large B‐cell lymphoma clinical trials? A CALGB 50303 analysis
Source: Cancer Med. 2023 Feb 17;12(7):8211–7. doi: 10.1002/cam4.5628 (PMC10134372; doi:10.1002/cam4.5628)
Supplement: Supplementary file 1 — Appendix S1 [file CAM4-12-8211-s001.docx]

**Supplemental Figure 1. Comparison of Local and Central iPET Status after Cycle 2 by VSS (n=106)** **after redefining 5-PS scores 1-2 as negative and scores 3-5 as positive on central reads**

| **Local Determination** | **Central Determination** | |  |
| --- | --- | --- | --- |
|  | **Positive** | **Negative** | **Total** |
| **Positive** | 40 | 12 | 52 |
| **Negative** | 22 | 32 | 54 |
| **Total** | 62 | 44 | 106 |
| *Agreement and performance measures using central reads as the reference.*  Overall Agreement: (40+32) / 106 = 67.9%  Positive Predictive Value: 40 / 52 = 76.9%  Negative Predictive Value: 32 / 54 = 59.3%  Sensitivity: 40 / 62 = 64.5%  Specificity: 32 / 44 = 72.7%  Kappa Statistic: 0.36 (95% CI: 0.19 - 0.53) | | | |

**Supplemental Figure 2. Progression free survival (PFS) and Overall survival (OS) landmarked at iPET, according to iPET status by 5-PS (negative= DS 1/2/3; positive= DS 4/5) (Figures S1A and S1B) and by prespecified ΔSUV groups (Figures S1C and S1D) using central PET interpretation (n=106)**

**Training for FDG-PET/CT Data Acquisition and Handling**

As part of the approval process, the Imaging Core Laboratory trained the technologists and responsible imaging physicians for the institutions in all aspects of patient handling, image acquisition, and image transfer. The Imaging Core Laboratory used a virtual site visit concept based on WebEx that could facilitate communication on any computer with Internet access. Outlined below is the WebEx conference information that was included in the institution-specific manual.

WebEx conferencing allowed the simultaneous display of images/text/help instructions (desktop presentations/desktop applications, such as Power Point) in a secure manner to participating sites to any computer with Internet access. For a WebEx conference, computers were required to have broadband Internet access and users must have had the authority to download a WebEx client. For another level of safety and quality, the Imaging Core Laboratory used a CALGB call-in number that was preferably used from a conference phone if there were more than one participant from any location.

The Imaging Core Laboratory created a video stream that included didactic information and training for technologists on any of the institution-specific issues. This information could be accessed at any time and/or downloaded at the institution for viewing. Furthermore, the Imaging Core Laboratory offered a virtual training course/workshop on an as-needed basis that could be attended by site personnel.

The Imaging Core Laboratory trained intra- and inter-institutional image readers using other independent data sets including imaging data import/export using the Workstations; image integrity/completeness check with the support of software tools; PET/CT fusion; ROI/VOI placements; SUV calculation.

The readers were benchmarked against a gold standard established for those images by a consensus assessment of the experienced nuclear medicine physicians selected by the Imaging Committee, to include the protocol Imaging Co-Chair. Readers were required to assess their consistency in assessment at least every six months. Furthermore, a web-based training module for nuclear medicine attendings reading trial studies was developed. The Imaging Core Laboratory did not provide basic skills, but a dedicated training on all assessment aspects of this trial.

**CALGB Imaging Core Lab Processing and Storage**

**HIPAA Compliance**

The image data sets were sent in a HIPAA-compliant manner. Upon receipt, the Imaging Core Laboratory verified that the images were de-identified and contained the trial and CALGB patient ID. In the case that they did contain patient private information, the Imaging Core Laboratory issued a non-compliance statement to the institution and de-identified the submitted data sets. The Imaging Core Laboratory worked in a completely de-identified manner and used study and patient identification alpha-numerics according to CALGB procedures.

**Quality Assurance and Quality Control of PET/CT Images**

During institutional approval, institution-specific manuals were established. All submitted PET/CT images were reviewed regarding technical specifications such as dosage, timing, acquisition, and reconstruction, and that they were compliant with the protocol. Any non-compliance or discrepancies were reported in the quality assessment and compliance review. This procedure was performed for every submitted data set and occurred within 72 hours upon receipt of the data set at the Alliance Imaging Core Lab at IROC Ohio. The quality of the PET/CT systems was verified on a daily and monthly basis. The review of the institution- and equipment-specific quality management, including daily quality assurance set-up and compliance to the policies, was established at the approval assessment and verified on an ongoing basis.

If, at the approval review of the institution, the Imaging Core Laboratory determined that there were insufficient quality standard operating procedures established for the site, it proposed that established procedures for the specific equipment type, based upon the manufacturer’s recommendation and established guidelines, be used. If at any time the institution was deemed to be non-compliant, a non-compliance statement was issued, and a remedy procedure suggested. If an institution did not remedy quality compliance concerns, the Imaging Core Laboratory revoked the approval of the institution. The institution then became ineligible for participation in the FDG-PET/CT companion until resolution of the defined issues. Imaging Core Laboratory guidelines were in alignment with the ACRIN guidelines.

The quality of each PET/CT image was assessed in three steps: 1) confirmation that images had been received and were electronically accessible; 2) image review for completeness and image quality by an experienced research associate or technologist who had access to staff nuclear medicine physicians in case of questions; and 3) review of images and post-processing results by an experienced staff member (nuclear medicine physician or radiologist). All ROI and calculation steps were electronically documented, and the quantitative data transferred into the trial database.

There were two components regarding readability of images. One was the technical readability (e.g., the images are in a DICOM format, which was a requirement, or in a data format of any of the three major equipment vendors: General Electric, Philips, and Siemens). The other aspect was the image quality. As described above, every data set received was reviewed regarding compliance to the acquisition and reconstruction protocol. Patient images that showed artifacts due to patient motion, extravasation of radiotracer, or other technical issues, including non-compliance of the timeline, were identified as such during the quality compliance review and reported as compliant/non-compliant or acceptable/unacceptable to the institution.

Submitted images were classified as “optimal” if they were compliant with all acquisition, reconstruction, and quality criteria without showing any artifacts. The classification “readable but not optimal” was reserved for those images which were compliant in the essential components, but were sub-optimal due to patient motion or non-detrimental deviation from the expected performance. The classification “not readable” was used for any other cases that cannot be characterized in the other two categories (i.e., no evaluable lesions; too much motion; wrong body location; data broken; images incomplete; non-DICOM image formats, such as JPEG images, bitmap images, screen-captured images, scanned films or hardcopies, etc.) and that were non-compliant.

An initial QA check was performed within 72 hours by the Imaging Core Laboratory upon receipt of each imaging study from the sites. The imaging studies were be stratified as (1) optimal, (2) readable but not optimal, and (3) not readable, based upon predetermined rules. Imaging studies that are not readable were communicated to the site and Imaging Co-Chair by the Imaging Core Laboratory and were encouraged to be immediately repeated. Imaging studies that were readable but not optimal were discussed with the Imaging Co-Chair to decide upon status in the analysis. It may be that these studies were included in the qualitative analysis, but not semiquantitative analysis.

Strict enforcement of compliance and quality assessment was necessary in order to ensure the highest quality of the data collected in this trial. The assessment of every submitted study and image within 72 hours of submission to the Imaging Core Laboratory regarding compliance and quality enabled not only the appropriate third-party review, but also a timely notification about non-compliance to the institution and the Alliance. The Imaging Core Laboratory copied non-compliance notices to the Alliance Imaging Committee and the Alliance Statistics and Data Center and supported the institution to ensure that compliance was achieved through appropriate measures. The Imaging Core Laboratory proposed remedy options that included expected timelines and revoked the institutional approval if compliance was not achieved within reasonable timelines. Any such action was coordinated with the Alliance Imaging Committee.

For non-compliant images: If images received by the Imaging Core Laboratory were not able to be analyzed (i.e., no evaluable lesions; data broken; images incomplete; non-DICOM image formats, such as JPEG images, bitmap images, screen-captured images, scanned films or hardcopies) and these potential problems could not be resolved, then the following scenarios applied:

a) If the problem occurred with the baseline images, then an imaging quality check report was sent to both the institution and the Alliance Imaging Committee Leadership, in which the Imaging Core Laboratory suggested that the baseline scans be rescheduled/reperformed, as well as that the imaging data be retransmitted. Otherwise, if the patient had been in follow-up studies, then the Alliance Imaging Committee Leadership would decide whether and how the patient could be included in the analyses.

b) If the problem occurred with the Cycle 2 images, but the baseline images were compliant, then an inquiry would be sent to the Alliance Imaging Committee Leadership to decide if the patient would be included in follow-up studies. If so, an imaging quality check report would be sent to both the institution and the Alliance Imaging Committee Leadership in which the Imaging Core Laboratory would remind the institution to avoid these problems in the follow-up scans and to provide DICOM-formatted imaging data for Cycle 6 and post-Cycle 6 images.

c) If the problem occurred with the Cycle 6 images, but both baseline and Cycle 2 images were compliant, then an inquiry would be sent to the Alliance Imaging Committee Leadership to decide if the patient would be included in any follow-up studies. If so, then an imaging quality check report would be sent to both the institution and the Alliance Imaging Committee Leadership in which the Imaging Core Laboratory would remind the institution to avoid these problems in post-Cycle 6 scans. Otherwise, if the Cycle 2 images were also non-compliant, then an imaging quality check report would be sent to both the institution and the Alliance Imaging Committee Leadership, and the Alliance Imaging Committee Leadership would decide if the patient will be excluded from further post-Cycle 6 PET/CT scans.

If the images received were analyzable, then an imaging quality check report would be sent to both the institution and the Alliance Imaging Committee Leadership, and the Alliance Imaging Committee Leadership would decide if the analysis for the patient was to be finished by the Imaging Core Laboratory.

Summary Reports: The Imaging Core Laboratory generated a monthly summary report for each institution that reported on the number of cases submitted, specification of images received, quality assurance failures and queries outstanding, and details of reconciliation processes in terms of images received/acquired, the quality/compliance assessment, etc. In addition to these institution-specific reports, the Imaging Core Laboratory created summary reports for the entire trial, including recruitment quality status and number of non-compliance queries. The Imaging Core Laboratory submitted quarterly and yearly reports to the Lymphoma FDG-PET Working Group (including experts from the NCI, FDA, Alliance, and PhRMA), the Imaging Co-Chair, and the Alliance Statistics and Data Center.

**Imaging Core Laboratory Image Reconstruction and Analysis**

Image reconstruction (i.e., an interactive reconstruction method with preference for OSEM reconstruction, 8 subsets, 2 iterations, followed by smoothing with a 6 mm 3-D Gaussian kernel) was performed using the manufacturer-recommended parameters and specified in the Technical PET Site Manual. Both visual/qualitative and semi-quantitative (SUV) PET data analysis were performed.

Both visual/qualitative and semi-quantitative (SUV) PET/CT data analysis were performed locally by the sites, as well as by the Alliance Imaging Committee in collaboration with the Imaging Core Laboratory. Data analysis of the study endpoints were based on readings by three qualified independent nuclear medicine physicians/radiologists, selected by the Imaging Committee. Readings were performed according to procedures outlined by the FDA imaging reports and consisted of two independent reads. Any discordance (defined by endpoints in the imaging analysis plan) was adjudicated by a third independent nuclear medicine physician/radiologist. The adjudicator could only select one of the two initial independent reads, as outlined by FDA independent read documentation. Quantitative assessments (e.g., tumor location, tumor size, ROI/VOI information, SUV, etc.) were electronically documented (screen captured from the Workstation) independently and used for determining consensus of readings, as well as inter-observer variability. Both Cycle 6 and Cycle 2 endpoints were dichotomous, and so were determined by consensus of two or three readings. If an eligible patient had a baseline PET/CT scan after the baseline excisional biopsy, the biopsied site was excluded from quantitative evaluation.

**Institutional & Imaging Core Laboratory Determination of Tumors and SUVs**

**Tumor Selection**: Up to six tumor masses > 2 cm were selected for analysis at baseline, and their locations and size (length and width) were defined. Tumor dimensions were determined in a single plane on the transverse CT image where the tumor appeared largest. The maximum dimension (length) was measured, followed by the width, which was perpendicular to the length. Tumor #1 was the tumor site with highest SUVmax; the others were selected in descending order of SUV. Location and dimensions of the index tumors were determined at baseline and at all subsequent PET/CT scans.

**SUVs:** SUVs for the study endpoints were calculated using decay-corrected administered dose and uncorrected body weight. SUVs were calculated at baseline and at all subsequent PET/CT scans. These procedures are described briefly below.

**Tumor SUVmax:** For the purposes of this study, the primary SUVs for calculation and reporting were the SUVmax within each of the index (up to 6) tumor volumes. This was determined by the nuclear medicine physician visually identifying the region or regions on the PET images that qualitatively appear to have the most intense FDG uptake and that correspond to known tumor based on other data (e.g., CT scan). SUVmax was determined on the transverse slice where the tumor visually appeared the brightest, as well as on several adjacent slices above and below, to be certain that the reported maximum was correct. Determinations were made on a 128 x 128 image matrix size image. VOIs could be placed where the tumor visually appears the brightest by 3-D ellipse/spherical or 3-D isosurface tools.

Prints of the image and the SUV cursor were acquired and retained for source documentation. As noted above, for the purposes of this protocol, up to six lesions were analyzed. The one with the highest SUVmax was reported as lesion #1; the others were identified in descending order of SUV.

**ABP SUVmean:** The ROI was placed on a transverse image or the spherical VOI with a 1 cm oval in the middle third of the aorta, well away from the edge. The mean SUV of the ROI/VOI was taken.

**Liver SUVmean**: The ROI was placed on a transverse image (a 3-5 cm long by 2 cm irregular region) or the spherical VOI (a 3-5 cm long by 2 cm irregular volume) through the mid liver with relative uniform intensities, which excluded the central hilum of the liver. The mean SUV of the ROI/VOI was taken.

**Other SUV**: As noted above, SUVs obtained and used for the endpoints of this study were based on body weight and were not corrected for body surface area or other measures of patient size/shape. However, patient height data was collected, and exploratory analysis of SUV corrected for body surface area and lean body mass planned to be performed to determine whether this correction provides more useful data than conventional, uncorrected SUV.
